# Integrating EEG biomarkers in the neurocognitive screening of executive functions in substance and behavioral addiction

**DOI:** 10.3389/fpsyt.2024.1472565

**Published:** 2024-09-19

**Authors:** Michela Balconi, Laura Angioletti, Davide Crivelli

**Affiliations:** ^1^ International research center for Cognitive Applied Neuroscience (IrcCAN), Università Cattolica del Sacro Cuore, Milan, Italy; ^2^ Research Unit in Affective and Social Neuroscience, Department of Psychology, Università Cattolica del Sacro Cuore, Milan, Italy

**Keywords:** substance use disorders, behavioral addictions, executive functions, EEG, wearable devices

## 1 EEG and executive functions across substance and behavioral addictions

Reward sensitivity and executive function (EF) impairment, including impaired decision-making and weak inhibitory control, are key characteristics observable at both behavioral and neural levels in addiction. These features are common to substance use disorder (SUD) and Gambling Disorder (GD), and to some extent, in Internet Gaming Disorder (IGD) (1). However, the research literature lacks consistency regarding these latter conditions and other behavioral disorders, necessitating further clarification.

Before, the Cortical Unbalance Model (2), which posits a left hemispheric imbalance in electrophysiological (EEG) delta, theta, and alpha cortical activity of the brain in addictive disorders, highlighted the significant role of both EEG markers and attitudes as potential precursors to this class of disorders (namely GD) with specific reference to reward sensitivity (3). On the other hand, several contributions stressed the role of Event Related Potentials (ERPs) as highly informative neurophysiological markers of neurocognitive impairment in addictive disorders, which can be useful starting from the diagnosis to cognitive rehabilitation and relapse prevention (4, 5). Such evidence showed how reward sensitivity and EFs impairment can be mapped with EEG markers in addiction. Understanding the neurocognitive impairment often accompanying addiction (6, 7) is crucial for developing effective assessment protocols and treatment strategies to enhance long-term therapeutic outcomes.

This opinion paper will discuss how these insights emphasize the need for an integrated addiction model that considers EEG correlates (both EEG frequency bands and ERPs) of impaired EFs, together with the evidence collected for the reward systems, and that guides neurocognitive screening procedures in SUD and behavioral addictions.

Indeed, it is argued that standardized procedures for the neurocognitive screening of EFs in patients with SUD and behavioral addiction could benefit from the inclusion of a set of computerized cognitive tasks with concomitant EEG data collection for determining the EEG correlates of EF impairment. Based on the EEG markers collected during the screening phase, and which can be continuously monitored even during daily life via wearable EEG devices, it will be possible to track neurocognitive impairment and its progress, as well as to propose personalized and targeted neurocognitive treatment approaches.

## 2 Concomitant collection of EEG markers during structured EF assessments

The recognition of cognitive impairments in addiction necessitates targeted assessment and intervention protocols. In a recent contribution, we have discussed how, despite the pivotal role of EFs dysfunction in influencing the clinical condition of SUD and behavioral addictions, a comprehensive understanding of the interrelationships and interdependencies among models of abuse, addiction-related neurofunctional changes, and specific patterns of neurocognitive/EF impairments remains a complex and largely unresolved matter (for an in-depth discussion on this subject, see (8). The identified contributing factors to these unresolved questions are:

- the absence of specialized assessment instruments designed to detect, qualify, and quantify the array of impaired higher cognitive functions in patients with addiction, considering its unique and frequently subtle manifestations.
- the lack of specific neurocognitive batteries with subtests dedicated to functions that are typically affected by addiction, such as inhibitory control.
- the peculiarity of the clinical subsamples with addiction, which are typically younger than reference clinical cohorts used to validate, screening tools designed for geriatric patients, and might present more subtle impairments, that require finer-grained evaluation (9, 10).

**Figure 1:**
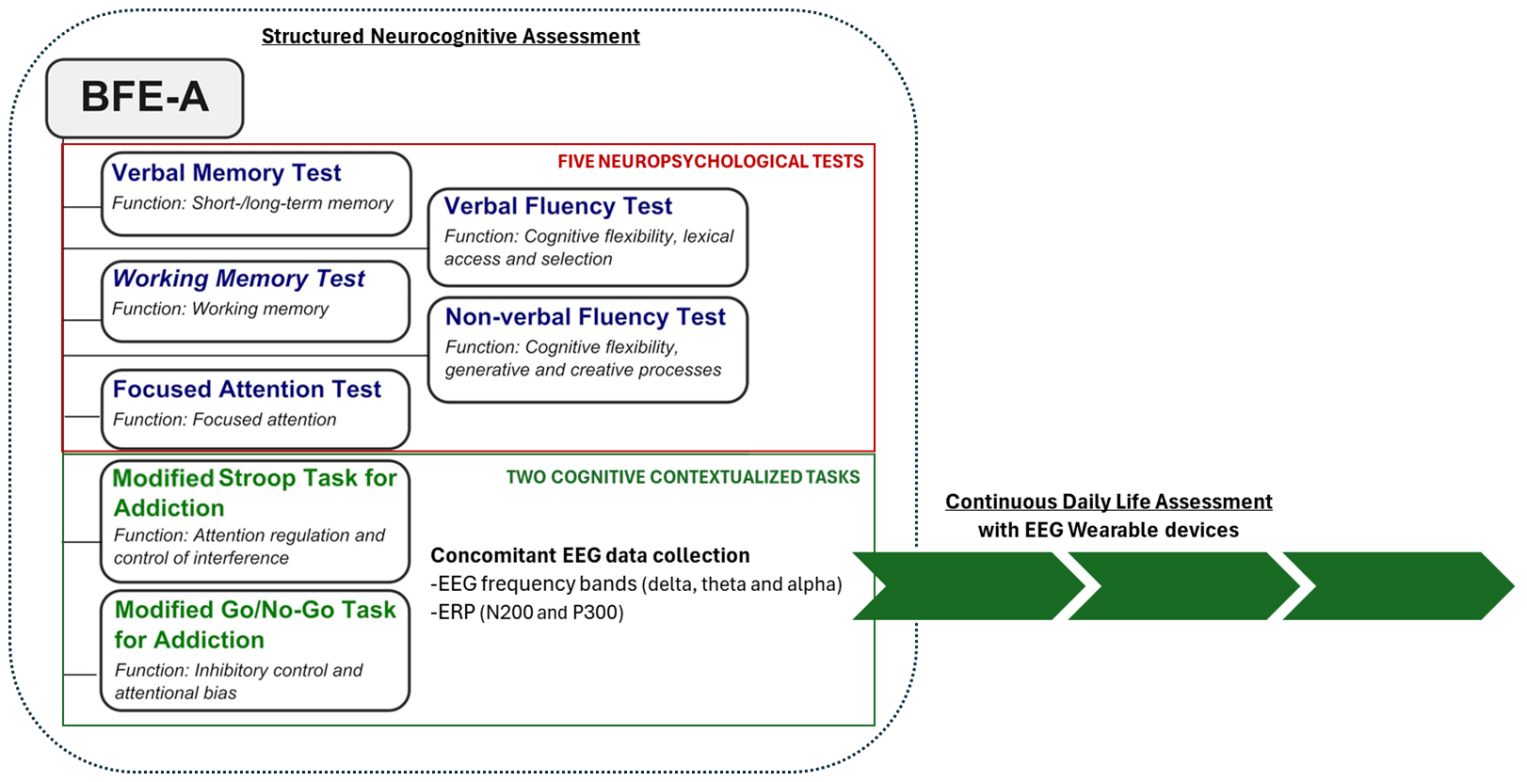
The figure illustrates the structure of BFE-A (retrieved and modified from 11) and outlines the proposal to collect EEG biomarkers during structured neurocognitive assessments, as well as to continuously monitor these biomarkers in daily life using wearable devices.

Given the need to reconceptualize the evaluation of addiction by proposing the assessment of EFs together with reward sensitivity in addiction, a new neurocognitive battery has been introduced (8). Indeed, recently, we developed a neurocognitive battery for the assessment of the executive functions in addiction (Battery for Executive Functions in Addiction – BFE-A, (8) for the Italian population, which encompasses five distinct neuropsychological tests and two tasks for the evaluation of the short- and long-term verbal memory, working memory, cognitive flexibility, focused attention, attention regulation and suppression of interference and inhibitory control (**Figure 1**). Specifically, the battery includes two contextualized tasks: a modified Stroop Task for Addiction (MST) and a Modified Go/No-go Task for Addiction (MGNT). The MST measures attention regulation and interference suppression, focusing on control of interference from addiction-related stimuli. The MGNT measures inhibitory control and attention bias suppression for addiction-related stimuli. Results in SUD populations suggest the BFE-A is a useful screening tool for addiction services, complementing the diagnostic process (12), though it lacks EEG marker integration.

An extensive body of literature highlighted the value of exploiting the concurrent EEG recording during classical or contextualized cognitive tasks for measuring attention regulation and response inhibition in SUD (13) and various paradigms, such as oddball, Go/No-Go, Stroop, Cue-reactivity, to name a few, are employed to tackle the neuronal correlates of different cognitive and affective processes in the brain (14).

Distinct EEG markers (both in the time and frequency domain) have been found to be related to EF processes during Stroop and Go/No-Go task. During the Stroop task, the frontal theta amplitude was found to be larger in the incongruent condition, as well as the occipital alpha desynchronization persisted longer in the same condition. While the evoked delta amplitude is larger in the congruent condition (15). In the Go/No-Go task, theta and delta bands separately increased in relation to response inhibition and were uniquely related to the N200 and P300 ERP components (16).

Considering the executive impairment in addictions and the EEG frequency bands regarded as reliable markers of EF, lower delta, theta, and slow alpha power in alcoholics during Go and NoGo response at the Go/NoGo task (17).

Individuals with vulnerability to internet addiction showed a reduction of alpha band in the left prefrontal cortex during a contextualized Go/NoGo Taks for both Go and No-Go conditions (18) IGD patients have been reported to experience raised resting state gamma and reduced beta and delta activity (19).

With specific reference to the response inhibition impairment and ERP, the N200 and the P300 components have been identified as key markers for alcohol disorders (4). Domínguez-Centeno and colleagues (20) demonstrated how the increased amplitude of the P3 component collected during a classical letter Go/No-Go task may be considered a useful endophenotype and a vulnerability marker to develop addictive behavior. During a “contextual Go/No-Go task” reduced amplitude and latency on the N2 component and increased P3 latency were found in a group of polydrug users compared to controls (21).

In gambling and gaming studies alterations in the NoGo N2 are described consistently (22) with initial evidence also for the NoGo-P3. However, Simkute and colleagues (23) argued that a vast heterogeneity regarding the EEG experimental paradigms being used, and the lack of clear guidelines and standardized procedures prevent the identification of measures capable of reliably discriminating or characterizing the population susceptible to addictive behavior or being able to diagnose and monitor these disorders.

Regarding IGD, recent reviews on EEG markers of neurocognitive response (24, 25) reported how EEG generally revealed reduced beta waves and increased theta bands in gaming disorder. IGD with depression demonstrated increased theta and decreased alpha waves. Whereas increased P300 was frequently associated with the impaired excessive allocation of attentional resources of the internet addiction disorder towards addiction-specific cues. IGD had increased whole brain delta waves at baseline, which a showed significant reduction post-therapy.

The integration of the assessment with EEG biomarkers proved to be useful for several reasons such as the distinction between the quality and quantity of cognitive impairment, as well as between different phases of the progress of the clinical condition, both in terms of severity (26) that of clinical course and detoxification (27). Moreover, this behavioral and neurocognitive integrated approach can even be extended to study other emerging addictive behaviors, such as addiction to physical exercise or binge eating disorder (28).

## 3 Route to the interventions: extensive monitoring and continuous assessment of neurophysiological markers

In advocating a holistic approach to mental health care, particularly in addressing addiction, we propose that effective management and treatment necessitate thorough monitoring and assessment of patients’ behaviors and psycho- and neuro-physiological responses both during computerized tasks included in structured neuropsychological evaluations (such as the BFE-A) as well as during ecological conditions.

Wearables, such as smartwatches, fitness trackers, and biosensors, have emerged as promising tools for continuous and objective data collection in healthcare. These devices, equipped with various sensors, enable the collection of data on physical activity, sleep patterns and can even measure chemical biomarkers like alcohol levels or drug metabolites, making them valuable for addiction-related monitoring.

In recent experimental applications, wearables are primarily utilized for monitoring substance abuse and craving. For instance, wearable sensors have been tested to track transdermal alcohol levels in real-time, offering insights into alcohol consumption patterns (29). Additionally, these wearables can detect drug use through sweat analysis, contributing to the early identification of relapse risk (30). Equipped with physiological sensors, wearables can also record changes in electrophysiological signals such as heart rate, skin conductance, and other relevant physiological data associated with gambling cravings or high-risk situations. This information proves valuable in identifying vulnerable episodes and implementing harm reduction strategies (31).

Building upon a valuable yet limited evidence base, we suggest the next challenge in enhancing monitoring, personalized assessment, and therapy for addiction is to refine neurocognitive assessment protocols. This involves measuring neural resource expenditure and effort through non-invasive physiological recordings. The advent of wearable technology has opened new possibilities for real-world cognitive load measurement, extending neuroassessment beyond research labs (32, 33). Monitoring cognitive load can provide critical insights into neurological health, cognitive disorders, and rehabilitation progress. Traditional methods of cognitive load assessment often rely on laboratory-based tests, self-reports, or observer ratings. Wearable technology may offer an integrative set of markers to complement observed performance metrics and more deeply assess the efficiency of neurocognitive processes.

For instance, wearables with EEG sensors have demonstrated their capability in monitoring cognitive load fluctuations in Alzheimer’s patients (34). Additionally, the potential of wearables like smartwatches to assess cognitive load during stroke rehabilitation exercises has been tested, aiding in therapy plan customization and cognitive recovery tracking (35). Wearable EEG, especially, has proven to be both usable and beneficial in monitoring cognitive effort during neurorehabilitation exercises, tailoring therapy plans for individuals recovering from neurological disorders (36). Although current evidence in mental health is limited, these neighboring studies suggest novel opportunities that could and should be validated and implemented soon.

Moreover, based on the EEG markers collected during the screening phase, will be possible to propose personalized and targeted neurocognitive treatment approach, such as neuromodulation interventions with neurofeedback devices or neurostimulation interventions with transcranial direct current stimulation techniques, which could promote greater neural functioning in both SUD and behavioral addiction.

As a final note, while it must be acknowledged that basic and applied research and validation of actually feasible remote monitoring protocols using wearables – especially in care of psychopathology – is still at its beginning, a few notes concerning strong points and challenges of such digital-health applications can already be pointed-out for further and coming discussion.

Among the most reported advantages:

- Wearables offer continuous and objective data, reducing reliance on unreliable self-reporting in addiction treatment. This impartial stream of data helps healthcare providers make informed decisions based on concrete evidence, overcoming issues like denial, stigma, or memory lapses.
- Continuous data collection enables the assessment of trends and patterns over time, facilitating personalized treatment adjustments. This is particularly valuable in addiction treatment, allowing therapists to observe patient progress, identify triggers, and modify treatment plans accordingly.
- Wearables allow for remote monitoring, supporting patients regardless of their location, especially beneficial for those in outpatient or telehealth programs. This promotes a comprehensive and continuous care model.
- Many wearables include gamification elements and feedback mechanisms, motivating individuals in recovery to stay committed to their treatment plans through goal setting, earning rewards, or receiving real-time feedback. This contributes to better treatment outcomes.
- R&D in monitoring addiction with wearables increasingly involve tighter integration with machine learning and AI algorithms. Analyzing complex data patterns enhances predictive capabilities, enabling earlier detection of relapse risks and adaptive interventions.

## 4 Conclusive remarks

The integration of such integrated approach and wearable technologies into existing healthcare systems can be challenging, requiring significant infrastructure investments and training for healthcare professionals. There are potential risks of over-reliance on technology, which might lead to reduced patient engagement and a lack of personalized care. Moreover, it also must be acknowledged that a few issues contrast such strong points and still raise practical and ethical concerns regarding such applications. Among them:

- Collecting sensitive data raises privacy and ethical concerns. It is essential to implement robust data protection measures, ensure informed consent, and maintain transparency regarding data use and sharing. Adequate data storage systems must be established.
- The accuracy of wearable data varies between devices and individuals, necessitating validation studies for trustworthy and clinically relevant data.
- Cost can be a barrier to access for specific patient populations or services, depending on psycho-social and geographic contexts.

Cognitive impairments in addiction are increasingly recognized as significant contributors to the persistence and relapse of this complex disorder. Both clinical research and practice call for informative neurocognitive and neurofunctional markers that could complement standard psychodiagnostics process. Future efforts in digital health R&D should capitalize current methodological, scientific, and ethical debate to promote feasible, accessible, and valuable solutions.
